# Soda-Water

**Published:** 1871-08

**Authors:** 


### SODA-WATER

Is delicious on a warm day, when great thirst is present; but the temptation to drink even more than is wanted, to prevent its being wasted, is such that many persons peril their lives in forcing more upon the stomach than Nature requires. Some soda-waters are more or less poisoned by coming in contact with the metallic vessels in which they are kept. When it is taken into account that in five minutes afterwards the body feels that the thirst is as thoroughly quenched by taking leisurely a glass of cold water, a wise care dictates the use of cold water in preference to the soda preparation.

